# Effect of hand reflexology in ameliorating anxiety, pain, and fatigue among patients undergoing coronary angiography

**DOI:** 10.1186/s12906-023-04256-6

**Published:** 2023-11-24

**Authors:** Eman Abdeen Ali, Wafaa Hassan Ali Awad, Mahmoud Abdelwahab Khedr, Emad Abd El Gawad Ali Rabie

**Affiliations:** 1 Medical Surgical Nursing Department, Faculty of Nursing, Alexandria University, Alexandria, Egypt; 2 Psychiatric and Mental Health Nursing, Psychiatric Nursing Department, Faculty of Nursing, Alexandria University, Shatebi, Bab Sharqi, Alexandria, Egypt

**Keywords:** Hand reflexology, Anxiety, Pain, Fatigue, Coronary angiography

## Abstract

**Background:**

Reflexology is a popular non-invasive complementary medicine technique to enhance anxiety, pain, and fatigue among patients undergoing coronary angiography.

**Objective:**

This study aimed to investigate the effect of hand reflexology in ameliorating anxiety, pain, and fatigue among patients undergoing coronary angiography.

**Methods:**

A quasi-experimental research design was used on 60 patients undergoing coronary angiography at Alexandria Main University Hospital's cardiology department (30 patients in each study and control group). Four tools were used to collect data: the socio-demographic and clinical data assessment sheet, the visual analogue scale, the Rhoten fatigue scale, and the Beck anxiety inventory.

**Results:**

Scores of moderate anxiety, intractable pain, and severe fatigue among the study group significantly decreased after 2 h and three days of applying hand reflexology.

**Conclusion:**

The current study findings showed that hand reflexology is a simple, non-invasive nursing intervention that is effective and useful for managing pain, fatigue, and anxiety in patients undergoing coronary angiography as it resulted in a significant reduction in the severity of pain, fatigue, and anxiety after coronary angiography in the study group compared to the control group.

**Trial registration:**

The study was registered in the clinical trial.gov database (Clinicaltrials.gov NCT05887362, 23/05/2023).

## Background

Invasive coronary angiography (ICA) continues to be the "gold standard" for the diagnisis of coronary artery disease (CAD) despite the development of non-invasive diagnostic tests [1]. The American Heart Association (AHA) declared that more than 13.2 million Americans have CAD, which results in an average yearly mortality rate of 1.2 million persons or myocardial infarctions [2]. According to the most recent WHO data, 173,871 deaths from CAD, or 32.40% of all fatalities, occurred in Egypt in 2020 [3].

Every time, information on the existence and severity of coronary artery disease (CAD) is needed to enhance patient symptoms or prognosis; therefore, the ICA is advised [4]. Local vascular problems, such as mild or significant hemorrhage, hematoma, pseudoaneurysm, and arteriovenous (AV) fistula, are widespread in the femoral artery area since it is typically used in the procedure for vascular access [5].

To reduce the risk of local vascular problems, total bed rest for around 6 h is typically advised following ICA. Long-term supine bed rest, in addition to the pain experienced during compression of the access channel, causes back pain and makes it challenging to perform physiological eliminations [6].

Compared to patients with other types of illnesses, cardiac patients generally have a greater incidence rate of anxiety [7]. More than 80% of cardiac patients are said to experience and show anxiety before CA [8]. Additionally, patients who have had many CA procedures report feeling as anxious as those who have only had one. Patients' past experiences, pain, stress, unfamiliar surroundings, fear of the unknown, the need for surgery, exposure to unfamiliar conditions, being physically apart from their loved ones, uncertainty surrounding the diagnosis, and concern about complications are the most common causes of anxiety in patients [2, 8].

## Significance of the study

Reflexology is a well-known, safe, and non-invasive alternative medicine technique. It alludes to a treatment applied to particular areas of the hands and feet. The compression on the hands or feet serves as a sensor linked to particular body areas. These sensors are activated by the reflexology technique to improve blood flow, energize, relax, and maintain homeostasis. It is one method for breaking the cycle of repetitive stress that people typically experience based on their lifestyle [9].

Reflexology is a nursing intervention shown in some research to increase immune system function, sleep quality, and quality of life while reducing pain, anxiety, tension, exhaustion, depression, nausea, and vomiting [10]. It may be helpful in treating chronic illnesses [11]. Moreover, reflexology influences pain perception and pain-impulse transmission by releasing endorphins [12]. Patients with various medical illnesses can benefit from hand reflexology by lessening their physical and emotional discomfort [13–15].

Reflexology intervention was used in many randomized controlled trials to examine the effectiveness of the treatment in patients with painful health conditions and diseases. Most studies have demonstrated reflexology's pain-relieving benefits [16]. Patients undergoing cardiovascular treatments or diagnostic procedures may benefit from reflexology to reduce their anxiety. A minimal number of higher-quality randomized controlled trials was conducted to assess the reflexology's efficacy in individuals who had recently undergone cardiovascular interventional procedures, and more research is required to create strong evidence and demonstrate the effectiveness of reflexology [17]. Moreover, it was previously recommended to conduct studies to address the beneficial effects of reflexology for cardiac patients [18]. Therefore, this study investigated the effect of hand reflexology in ameliorating anxiety, pain, and fatigue among patients undergoing coronary angiography.

## Subjects and methods

### Study design and setting

A quasi-experimental research design was utilized in this study. It was conducted at the Cardiology Department, Alexandria Main University Hospital. The study was registered in the clinical trial.gov database (Clinicaltrials.gov NCT05887362, 23/05/2023). Available at: https://ichgcp.net/clinical-trials-registry/NCT05887362

### Participants

The sample size was determined using the G*Power Windows 3.1.9.7 program, with the following criteria: effect size = 0.25, power (1-err prob) = 0.85, err α prob = 0.05, number of groups examined = 2, and the number of measurements = 3. Thus, the study group included 30 patients scheduled for CA, and the control group had an equal number. The inclusion criteria were patients between the ages of 20 and 60 who were scheduled for non-emergency CA, absence of any hands’ sensory motor disorders or upper limb vascular injuries, no abnormalities such as amputations, burns, skin lesions, and intervertebral disc herniation, and no history of mental illnesses.

**Fig. 1:**
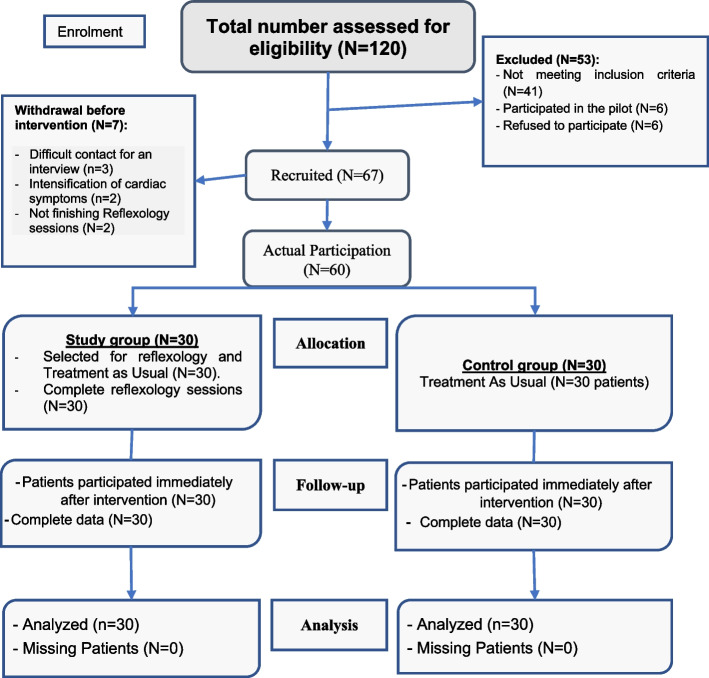
Flow chart for recruiting the study participants

As the diagram (Fig. 1) revealed, patients were selected from the cardiology department (N = 120) for reflexology sessions over four months. The number of patients excluded from the total assessed for eligibility (*N* = 53). Of them, 41 patients did not meet the inclusion criteria, six were excluded due to participation in the pilot study, and six refused to participate in reflexology sessions. Furthermore, seven patients withdrew prior to intervention due to the severity of their cardiac symptoms, the difficulty in contacting them for an interview, and their decision not to complete the sessions. All patients who took part in the study and control groups were treated as usual (TAU); the patients who were selected for the study group participated in reflexology sessions. All study and control groups completed the measurements after 2 h and three days after the intervention (*N* = 60).

### Data collection tools

Four tools were used for collecting the data in this study:

- *Tool I*: The socio-demographic and clinical data assessment sheet was created by the researcher to identify patients’ socio-demographic and clinical data, including their name, age, sex, marital status, level of education, occupation, area of residence, medical diagnosis, associated diseases, previous hospitalization and its reasons, history of smoking, previous history of CA, medications, and family history of CA.
- *Tool II*: Visual Analogue Scale (VAS), developed by [19] to assess pain intensity. It is a 10 cm horizontal line with two ends; the left end typically denotes "no pain," while the right end typically denotes "worst possible pain." On the line, the patient was told to mark the location of the current pain. Score system: 0 represents "no pain," 1–3 "mild pain," 4–7 "moderate pain," 8–9 "severe pain," and 10 represents the "worst intractable suffering."
- *Tool III*: The Rhoten Fatigue Scale (RFS) is a subjective rating scale developed by [20] to measure a patient’s fatigue level. It comprises a 10-cm line with highly positive and negative statements at either end. Scores ranged from 0 to 10 for positive and negative fatigue phrases. It is appropriate to interpret the scale's results as follows: 0 (no fatigue), 1–3 (mild), 4–6 (moderate), 7–9 (severe), and 10 (extreme fatigue).

- *Tool IV*: The Beck Anxiety Inventory (BAI) was developed in 1988 by [21] to assess the severity of anxiety symptoms. The Likert scale for the 21 items that comprise the BAI ranges from 0 to 3. The overall score is between 0 and 63. The BAI scores are divided into four categories: minimal anxiety (0–7), mild anxiety (8–15), moderate anxiety (16–25), and severe anxiety (26 to 63)

### Data collection and analysis

#### Administrative steps

The responsible authorities of the Faculty of Nursing, Alexandria University, including the Research Ethics Committee, were permitted to conduct this study. Official permission was received from the head of the Cardiology Department at the Main University Hospital after an explanation of the aim of the study.

#### Preparation phase

Tools II, III, and IV were translated into Arabic and reviewed by bilingual medical-surgical and psychiatric nursing experts. Also, a jury of five medical-surgical and psychiatric nursing experts was selected to test the face and content validity of the translated tools.

#### Pilot study and reliability

To test the clarity and applicability of the research tools, a pilot study including six patients, or 10% of the sample, was conducted. Modifications were made as necessary, such as "complications of previous angiography," which were omitted from tool I because patients could not answer this question and were not included in the patient's medical records. The clarity of other tools was ensured, especially after translation into Arabic. They were not included in the study's actual sample of participants. The study tools' internal consistency was assessed using Cronbach's alpha test. Tool II (= 0.824), Tool III (= 0.788), and Tool IV (= 0.868) were reliable.

#### Actual study

Patients’ medical charts in the department were reviewed to identify those who meet the inclusion criteria. Patients who met the predetermined inclusion criteria were recruited for the study through convenience sampling. Participants were divided into two equal groups (the control and the study groups). The researchers started by introducing themselves, explaining the purpose of the study, and then taking informed written consent from those who agreed to participate.

On the day of coronary angiography, after the CA procedure, and before implementing hand reflexology intervention for the study group, all patients in the control and study groups were interviewed individually to identify their socio-demographic and clinical data using Tool I and to assess their level of pain, fatigue, and anxiety using Tools II, III, and IV.

The control group was selected first and received the routine care delivered by the hospital, such as assessment of vital signs, supine position, complete bed rest, and hygienic care. They didn’t receive the hand reflexology intervention.

## For the study group

Initially, one of the researchers undertook a 5-weeks certified training course in reflexology at the faculty of physical education for girls (Licence: 44580/2019). Then, the other researchers received training to conduct hand reflexology sessions for patients in the study group. They ensured that the location where the meetings were held was peaceful, well-lit, organized, motivating, and pleasant to increase patients’ compliance with the sessions. Male patients received hand reflexology from a male researcher, while female patients received it from a female researcher as patients were embarrassed and anxious when touched by a researcher of a different gender during reflexology application due to cultural and religious factors. Anxiety is one of the study variables, so the patient should feel comfortable.

The steps of reflexology, its sequence, and the duration for each step were standardized as the senior researcher supervised the other researchers simultaneously to ensure consistency of application of reflexology principles for all participants. A well-defined protocol for reflexology application that is standardized across all researchers involved in the study. This protocol included detailed instructions on the techniques, pressure points, duration, and any other relevant factors to ensure consistency in the intervention. Further, regular training sessions and practice opportunities were delivered to the researchers to improve their skills and guarantee uniformity in the delivery of reflexology. Each patient in the study group received eight hand reflexology sessions after CA in a room, separating them from the patients in the control group. Each session lasted 10 min, followed by a 5-min rest period.

Using pleasant, odorless almond oil to lubricate the patients' hands, the researcher used Ingham's approach to hand reflexology for 10 min, applying pressure for 5 min on the right hand and then the left. The entire palm was first subjected to pressure for two minutes. Each organ and body part has corresponding reflex points on the hands. Three pressure points were used to simulate the solar plexus, heart, and pituitary. The location of the correct, corresponding areas of the solar plexus, heart, and pituitary was determined by using a reflexology chart. For each reflex point, the pressure was applied using the thumb-walking technique, whereby the fully extended thumb was set on the reflex point. Then, flexing the first thumb knuckle upward and moving the thumb knuckle up and down gradually and steadily, walking it over the reflex point while keeping ongoing pressure. After that, a circular thumb rotation is used on the reflex point while slowly boosting pressure without provoking pain or discomfort.

Evaluation: After 2 h of CA for both the control group and the study group (after receiving eight sessions of hand reflexology), the researcher re-evaluated the degree of pain, fatigue, and anxiety in both groups after three days of CA while the patients are coming for follow-up. The data was collected between July 1, 2022, and November 15, 2022.

*Statistical Analysis:* Data were entered into the Statistical Package for Social Sciences (SPSS) 25.0 V program for analysis. The Kolmogorov–Smirnov (KS) tests were used to ensure the normality of the collected data. The numbers and percentages were utilized for the description of the qualitative data. The chi-square test was employed for categorical variables to compare results across different groups. When more than 20% of the cells have an anticipated count lower than five, Fisher's Exact or Monte Carlo correction was applied to correct the chi-square statistic. The student t-test was utilized for normally distributed quantitative data to compare the two groups under study. The Friedman test compared more than two periods or stages and abnormally distributed quantitative data. The significance levels were obtained at *p*-values of ≤ 0.05.

## Results

### Demographic and clinical characteristics of the participants

Table 1 reveals that 46.7% of the study group and 60% of the control group were aged between 40 to less than 50 years. More than half of the patients in the study and control groups were males and married. Also, 70% of patients in the study group and 63.3% in the control group lived in urban areas. Patients who had completed secondary education represented 30% of the study group and 43.3% of the control group. The absence of any statistically significant difference between the study and control groups indicated that both groups are almost matched.

**Table 1:** Socio demographic characteristics of patients in study and control groups (*n* = 60)

Socio-demographic	Control (*n* = 30)		Study (*n* = 30)		Test of Sig	p
	**No**	**%**	**No**	**%**		
**Age**						
< 40	2	6.7	4	13.3	χ^2^ = 1.348	^MC^*p* = 0.525
40- < 50	18	60.0	14	46.7		
50–60	10	33.3	12	40.0		
Min. – Max	38.0 – 56.0		35.0 – 60.0		t = 0.732	0.468
Mean ± SD	44.97 ± 4.79		46.17 ± 7.60			
Median	44.0		44.0			
**Sex**						
Male	16	53.3	18	60.0	χ^2^ = 0.271	0.602
Female	14	46.7	12	40.0		
**Marital status**						
Single	2	6.7	0	0.0	5.300	^MC^*p* = 0.120
Married	21	70.0	19	63.3		
Divorced	3	10.0	9	30.0		
Widow	4	13.3	2	6.7		
**Residence**						
Urban	19	63.3	21	70.0	0.300	0.584
Rural	11	36.7	9	30.0		
**Level of education**						
Illiterate	4	13.3	8	26.7	2.218	0.528
Read and write	8	26.7	7	23.3		
Secondary	13	43.3	9	30.0		
High education	5	16.7	6	20.0		
**Occupation**						
Manual work	17	56.7	15	50.0	1.002	0.606
Cleric work	9	30.0	8	26.7		
Hard work	4	13.3	7	23.3		

*χ*^*2*^ Chi square test, *MC* Monte Carlo, *SD* Standard deviation, *t* Student t-test

*P p* value for comparing between the studied groups

Table 2 shows that heart diseases and hypertension were the most common among the studied patients. Previously hospitalized patients represented 56.7% and 50% of the study and control groups, respectively. Most patients in the study group (83.3%) and control group (90%) had a smoking history. In addition, 56.7% of patients in the study group and 66.7% of patients in the control group had no previous history of CA. Also, an equal percentage (60%) of patients in the study and control groups had no family history of angiography. Both prescribed and over-the-counter medications constituted 70% and 53.3% of medications taken by patients in the study and control groups, respectively.

**Table 2:** Clinical characteristics of patients in study and control groups (*n* = 60)

Clinical data	Control (*n* = 30)		Study (*n* = 30)		χ^2^	p
	**No**	**%**	**No**	**%**		
**Associated diseases**						
Hypertension	8	26.7	9	30.0	0.772	^MC^*p* = 0.902
Diabetes	6	20.0	8	26.7		
Heart diseases	12	40.0	10	33.3		
Other	4	13.3	3	10.0		
**Previous hospitalization**						
Yes	15	50.0	17	56.7	0.268	0.605
No	15	50.0	13	43.3		
**History of smoking**						
Yes	27	90.0	25	83.3	0.577	^FE^*p* = 0.706
No	3	10.0	5	16.7		
**Previous history of coronary angiography**						
Yes	10	33.3	13	43.3	0.635	0.426
No	20	66.7	17	56.7		
**Family history of coronary angiography**						
Yes	12	40.0	12	40.0	0.000	1.000
No	18	60.0	18	60.0		
**Medication history**						
Prescribed	7	23.3	5	16.7	1.827	0.401
Over the counter	7	23.3	4	13.3		
Both	16	53.3	21	70.0		

*χ*^2^ Chi square test, *MC* Monte Carlo, *FE* Fisher Exact, *SD* Standard deviation, *t* Student t-test

*P p* value to compare between the studied groups

### Anxiety among the study and control groups

Table 3 reveals that the scores of moderate anxiety levels among the study group significantly decreased after 2 h and three days of applying hand reflexology (values before = 33.3%, after 2 h = 13.3%, and after three days = 16.7%). Regarding the control group, it was found that moderate anxiety was increased after 2 h and after three days of CA (values before = 26.7%, after 2 h = 30%, and after three days = 30%). Moreover, the current table also illustrates that severe anxiety levels decreased in both the study and control groups after 2 h and after three days. The table also reveals a significant difference among both groups after 2 h and three days after implementing the intervention (*p* = 0.003 and 0.034, respectively).

**Table 3:** Scores of "anxiety" among the study and control groups after CA before the reflexology intervention, after 2 h and after 3 days of the intervention

Anxiety	Control (*n* = 30)						Study (*n* = 30)						Test of sig. (p_1_)	Test of sig. (p_2_)	Test of sig. (p_3_)
	**Before**		**After 2 h**		**After 3 days**		**Before**		**After 2 h**		**After 3 days**				
	**No**	**%**	**No**	**%**	**No**	**%**	**No**	**%**	**No**	**%**	**No**	**%**			
**Anxiety Level**															
Minimal anxiety	3	10.0	6	20.0	6	20.0	0	0.0	15	50.0	17	56.7	3.243 (^MC^*p* = 0.393)	13.934^*^ (0.003^*^)	8.696^*^ (0.034^*^)
Mild anxiety	5	16.7	4	13.3	8	26.7	7	23.3	9	30.0	5	16.7			
Moderate anxiety	8	26.7	9	30.0	9	30.0	10	33.3	4	13.3	5	16.7			
Severe anxiety	14	46.7	11	36.7	7	23.3	13	43.3	2	6.7	3	10.0			
**Fr.(p**_**0**_**)**	**3.310 (0.191)**						30.198^*^ (< 0.001^*^)								

*χ*^*2*^ Chi square test, *MC* Monte Carlo, *FE* Fisher Exact, *SD* Standard deviation, *t* Student t-test, *Fr* Friedman test

p_1_: “*p* value” to compare between the groups studied before intervention period

p_2_: “*p* value” to compare between the groups studied after 2 h period

p_3_: “*p* value” to compare between the groups studied after 3 days period

p_0_: “*p* value” to compare between the studied periods in each group

^*^Statistically significant at *p* ≤ 0.05

### Pain among the study and control groups

Table 4 illustrates that the scores of intractable pain among the study group were significantly decreased after 2 h and after three days of applying reflexology (values before = 36.7%, after 2 h = 10%, and after three days = 0%). In comparison to the control group, it could be noticed that the intractable pain was slightly decreased after 2 h, then increased again after three days (values before = 20%, after 2 h = 16.7%, and after three days = 23.3%). The table denotes a significant difference between both groups after 2 h and three days of implementing the reflexology intervention (p < 0.05).

**Table 4:** Scores of "pain" among the study and control groups after CA before the reflexology intervention, after 2 h and after 3 days of the intervention

Pain	Control (*n* = 30)						Study (*n* = 30)						Test of sig. (p_1_)	Test of sig. (p_2_)	Test of sig. (p_3_)
	**Before**		**After 2 h**		**After 3 days**		**Before**		**After 2 h**		**After 3 days**				
	**No**	**%**	**No**	**%**	**No**	**%**	**No**	**%**	**No**	**%**	**No**	**%**			
**Pain level**															
**No pain**	1	3.3	2	6.7	2	6.7	2	6.7	5	16.7	11	36.7	4.176 (^MC^*p* = 0.405)	11.779^*^ (^MC^*p* = 0.015^*^)	22.933^*^ (< 0.001^*^)
**Mild**	3	10.0	3	10.0	2	6.7	4	13.3	10	33.3	10	33.3			
**Moderate**	5	16.7	9	30.0	9	30.0	5	16.7	10	33.3	6	20.0			
**Severe**	15	50.0	11	36.7	10	33.3	8	26.7	2	6.7	3	10.0			
**Intractable pain**	6	20.0	5	16.7	7	23.3	11	36.7	3	10.0	0	0.0			
**Fr.(p**_**0**_**)**	**2.886 (0.236)**						36.200^*^ (< 0.001^*^)								

χ^2^: Chi square test, *MC* Monte Carlo, *FE* Fisher Exact, *SD* Standard deviation, *t* Student t-test, *Fr* Friedman test

p_1_: “*p* value” to compare between the groups studied before intervention period

p_2_: “*p* value” to compare between the groups studied after 2 h period

p_3_: “*p* value” to compare between the groups studied after 3 days period

p_0_: “*p* value” to compare between the studied periods in each group

^*^Statistically significant at *p* ≤ 0.05

### Fatigue among the study and control groups

Table 5 reveals that the scores of severe fatigue levels among the study group significantly decreased after 2 h and three days of applying reflexology (values before = 33.3%, after 2 h = 10%, and after three days = 6.7%). On the other hand, it could be noticed that the severe fatigue level in the control group was slightly decreased after 2 h, then increased again after three days (values before = 43.3%, after 2 h = 36.7%, and after three days = 46.7%). The table reveals a significant difference among both groups after 2 h and after three days from applying the reflexology intervention (*p* = 0.001) during the two periods.

**Table 5:** Scores of "fatigue" among the study and control groups after CA before the reflexology intervention, after 2 h and after 3 days of the intervention

	**Control (*n* = 30)**						**Study (*n* = 30)**						**Test of sig. (p**_**1**_**)**	**Test of sig. (p**_**2**_**)**	**Test of sig. (p**_**3**_**)**
	**Before**		**After 2 h**		**After 3 days**		**Before**		**After 2 h**		**After 3 days**				
	**No**	**%**	**No**	**%**	**No**	**%**	**No**	**%**	**No**	**%**	**No**	**%**			
**Fatigue**															
**Lack of fatigue**	0	0.0	2	6.7	2	6.7	2	6.7	6	20.0	15	50.0	3.878 (^MC^*p* = 0.445)	17.797^*^ (^MC^*p* = 0.001^*^)	21.941^*^ (< 0.001^*^)
**Mild**	4	13.3	3	10.0	6	20.0	4	13.3	15	50.0	9	30.0			
**Moderate**	5	16.7	8	26.7	6	20.0	9	30.0	4	13.3	4	13.3			
**Severe**	13	43.3	11	36.7	14	46.7	10	33.3	3	10.0	2	6.7			
**Very severe fatigue**	8	26.7	6	20.0	2	6.7	5	16.7	2	6.7	0	0.0			
**Fr.(p**_**0**_**)**	**4.490 (0.106)**						30.299^*^ (< 0.001^*^)								

χ^2^: Chi square test, *MC* Monte Carlo, *FE* Fisher Exact, *SD* Standard deviation, *t* Student t-test, *Fr* Friedman test

p_1_: “*p* value” to compare between the groups studied before intervention period

p_2_: “*p* value” to compare between the groups studied after 2 h period

p_3_: “*p* value” to compare between the groups studied after 3 days period

p_0_: “*p* value” to compare between the studied periods in each group

^*^Statistically significant at *p* ≤ 0.05

## Discussion

With stressful events and interventions like CA, the level of anxiety may increase, resulting in physiological and psychological complications. Nurses have an essential role in decreasing patients’ fear and anxiety. The results of this study illustrated that a significant difference was found between the study and control groups after implementing the reflexology program. Moderate and severe anxiety scores were lower in the study group in all evaluation periods after the intervention than in the control group. It could be due to massage and nerve stimulation during reflexology, which results in relaxation, alleviate stress, improve blood circulation, and restore body balance. In addition, touching the patient’s skin during reflexology stimulates the release of endogenous endorphins, which reduce anxiety, stress, and pain [22].

This finding is consistent with Kahraman, 2019 [23], who found that the anxiety level of patients undergoing CA was significantly reduced by reflexology intervention. Furthermore, the current results were supported by Amer et al., 2022 [24], who reported a significant decrease in mean scores of anxiety in the study group of patients undergoing CA immediately and after 30 min of applying foot reflexology because the pressure on the reflex sites in the feet can impact target organ performance and function, resulting in inducing and promoting relaxation. In addition, several studies revealed that reflexology massage contributes positively to anxiety in cardiovascular interventional procedures [17, 25, 26].

The current study results illustrated that the pain severity was significantly reduced in the study group after 2 h and three days of hand reflexology intervention compared to the control group. This finding can be rationalized in accordance with gate control theory, as reflexology is a standard method of massage that stimulates the large nerve fibers, resulting in blockage of pain signal transmission and inhibiting the central nervous system from receiving it. In addition, reflexology and massage efficiently reduce pain because the muscles are relaxed, the blood flow is increased, and metabolic waste products are removed.

This finding is consistent with the findings of Kardan et al., 2020 [27], who discovered that the intensity of back pain after CA was significantly reduced in the study group after applying foot reflexology intervention compared to the control group. In addition, it is in line with Babadi et al., 2016 [28], who found a significant difference after the reflexology intervention for low back pain between the study and control groups, as pain perception and intensity in the study group were lower than in the control group. This finding is also supported by Taman et al., 2018 [29], who concluded that cardiac catheterization patients reported a lower pain intensity after receiving a foot massage.

The results of this study provide additional evidence supporting the potential of reflexology to decrease fatigue in patients undergoing medical procedures effectively. The current study results showed a statistically significant decline in the level of fatigue, especially moderate and severe fatigue levels, in the study group compared with the control group. It may be due to the physiological changes caused by reflexology, which increase the patient's sense of well-being and comfort. Furthermore, massage and pressure during reflexology intervention enhance blood circulation, which removes accumulated lactic acid from the muscles, reducing muscular stress and fatigue [30].

This finding is consistent with Rejeh et al., 2020 [14], who found that hand reflexology effectively reduced fatigue severity after CA, as the study group reported lower fatigue levels after receiving hand reflexology than the control group. It is also supported by Rambod et al., 2019 [11], who found that foot reflexology effectively decreased fatigue in patients with lymphoma, and Nour Mohammadi et al., 2019 [31], who reported a significant decrease in fatigue level and severity in patients who had breast cancer after receiving the reflexology intervention. Moreover, Özdemir et al., 2013) concluded reflexology positively affected hemodialysis patients' fatigue [32].

## Conclusion

The current study findings showed that hand reflexology is a simple, non-invasive nursing intervention that is effective and useful for relieving and ameliorating pain, fatigue, and anxiety in patients undergoing CA, as it resulted in a significant reduction in the severity of pain, fatigue, and anxiety after CA in the study group compared to the control group.

## Recommendations

Based on the study results, it is recommended to include hand reflexology in the overall nursing care provided for patients undergoing CA. Establishing training programs and workshops periodically and regularly for nurses is needed to upgrade their knowledge and skills regarding hand reflexology. Moreover, it is essential for the hospital's management systems to motivate and support nurses in applying hand reflexology for patients undergoing CA. To obtain more generalizable data, a replication of this study using a larger sample drawn from other Egyptian regions is advised.

## Limitations

The findings’ limitations include the following: they are less generalizable due to the small sample size and the fact that only one region of Egypt was used for the sample. The novelty of the intervention, lack of experience and knowledge about it, the decreased number of highly educated patients, and the cultural variations between them affected some patients' decisions to participate in the study. A lack of data exists regarding the long-term effects of hand reflexology on pain, fatigue, and anxiety.

Therefore, further longitudinal follow-ups are required in future research. Despite these limitations, the study showed exciting findings on the significant effect of hand reflexology in ameliorating anxiety, pain, and fatigue among CA patients.

## Data Availability

The datasets used or analyzed in this study are available from the corresponding author upon request.
